# Continuous 4‐week ECG monitoring with adhesive electrodes reveals AF in patients with recent embolic stroke of undetermined source

**DOI:** 10.1111/anec.12649

**Published:** 2019-05-02

**Authors:** Tuomas J. Lumikari, Jukka Putaala, Anne Kerola, Gerli Sibolt, Jani Pirinen, Sami Pakarinen, Mika Lehto, Tuomo Nieminen

**Affiliations:** ^1^ Department of Neurology Helsinki University Hospital Helsinki Finland; ^2^ Department of Internal medicine Päijät‐Häme Central Hospital Lahti Finland; ^3^ Department of Clinical Physiology and Nuclear Medicine HUS Medical Imaging Center, Helsinki University Hospital, University of Helsinki Helsinki Finland; ^4^ Department of Cardiology, Heart and Lung Center Helsinki University Hospital Finland

**Keywords:** atrial fibrillation, electrocardiogram, embolic stroke of undetermined source, stroke

## Abstract

**Background:**

Atrial fibrillation (AF) frequently escapes routine stroke workup due to its unpredictable and often asymptomatic nature, leaving a significant portion of patients at high risk of recurrent stroke. Recent trials emphasized continuous electrocardiogram (ECG) monitoring in the detection of occult AF. We screened AF in patients meeting the embolic stroke of unknown source (ESUS) criteria using an external miniaturized recorder with an adhesive electrode.

**Methods:**

Patients aged ≥50 with recent ESUS were prospectively screened and assigned to wear a 1‐lead ECG device capable to record continuous ECG for up to 4 weeks. Electrodes were replaced every 3–4 days. Primary outcome was proportion of patients completing at least 80% of monitoring. Secondary outcome measures included incidence of AF and initiation of oral anticoagulation therapy after AF detection.

**Results:**

Fifty‐seven patients were monitored (mean age 64.5 ± 8.2 years, median delay from stroke to the start of monitoring 8 days, IQR 4–44). Of these, 51 patients (89.5%) completed at least 80% of the desired monitoring period. We detected AF ≥30 s in seven patients (12.3%), all of whom initiated anticoagulation therapy. Atrial fibrillation was revealed in six patients (85.7%) within the first week of monitoring. Compared to patients without AF, patients with AF were older (70.6 ± 5.1 vs. 63.6 ± 8.3 years, *p* < 0.011) and more obese (body mass index 30.0 ± 3.4 vs. 26.6 ± 4.6, *p* < 0.039).

**Conclusions:**

Prolonged ECG monitoring with an external device using adhesive electrodes is feasible in ESUS patients, since nine out of ten patients used the device appropriately and AF was detected in one out of eight patients.

## 1 INTRODUCTION

Ischemic stroke is one of the leading causes of mortality and long‐term disability (Benjamin et al., 2017). Among the severest and most disabling strokes are those with underlying atrial fibrillation (AF) (Kolominsky‐Rabas, Weber, Gefeller, Neundoerfer, & Heuschmann, 2001; Lamassa et al., 2001). Atrial fibrillation is a common arrhythmia and a well‐known risk factor for stroke (Wolf, Abbott, & Kannel, 1991). Those with AF and a previous stroke history represent the group with by far the highest risk of future stroke (Piccini et al., 2013). The risk of recurrent stroke can, however, be efficiently decreased by oral anticoagulation therapy (Ntaios, Papavasileiou, Diener, Makaritsis, & Michel, 2017).

Secondary preventive treatment after stroke is determined by stroke pathogenesis. However, pathogenesis remains unknown in 25%–30% of all ischemic strokes despite complete diagnostic evaluation, leading to diagnosis of cryptogenic stroke. A more sophisticated classification of cryptogenic strokes with an embolic neuroimaging pattern—embolic stroke of undetermined source (ESUS)—was recently introduced and defined as a nonlacunar stroke without a major cardioembolic source nor major arterial occlusions or other causes for the stroke (Hart, Catanese, Perera, Ntaios, & Connolly, 2017). Patients in the ESUS subgroup suffer from a high stroke recurrence risk, comparable to those with AF and other known high‐risk sources of embolism (Putaala et al., 2015). Notably, a good proportion of ESUS patients may harbor undiagnosed AF (Wachter & Freedman, 2017). Early detection of AF is, in turn, important as delayed diagnosis of AF after stroke itself increases the risk of recurrent stroke (Chou et al., 2017).

Standard methods of diagnosing AF after stroke include admission and repeat 12‐lead electrocardiograms (ECGs), continuous inpatient ECG and telemetry monitoring, or Holter ECG recording lasting for 24 to 72 hr. This routine diagnostic workup is ineffective due to arrhythmias’ paroxysmal nature with often unpredictable, asymptomatic onset, and only about 5% of patients are diagnosed with new AF (Sposato et al., 2015). These factors, alongside with poor ECG quality due to lead loss and lack of patient compliance with wearing the monitoring device, have been suspected to lower detection rates of AF in patients with stroke.

Recent randomized trials showed the value of continuous ECG monitoring in the detection of occult AF in stroke patients using either external loop recorders (Gladstone et al., 2014), implantable cardiac monitors (Sanna et al., 2014), or 7‐day Holter ECG (Wachter et al., 2017). Detection rates of newly diagnosed AF from 0% up to 25% in 1‐ to 4‐week monitoring were reported (Silverman, 2016; Sposato et al., 2015; Wachter et al., 2017). Increasing monitoring length beyond 4 weeks (Silverman, 2016) or conducting multiple intermittent monitoring periods (Wachter et al., 2017) yielded only little addition compared to a shorter monitoring period. To our knowledge, so far only a single study enrolling ESUS patients has been conducted, reporting AF detection rate of 24% in 13 months with an implantable cardiac monitor (Israel et al., 2017). ECG devices using adhesive electrodes or belts with one to three lead ECG have been successfully used and with high patient compliance rates among various other groups of arrhythmia patients (Ackermans et al., 2012; Engel, Mehta, Fogoros, & Chavan, 2012; Rosenberg, Samuel, Thosani, & Zimetbaum, 2013).

We aimed to screen AF in patients meeting ESUS criteria and to evaluate patient adherence to a light‐weighted one‐lead ECG device using changeable adhesive electrodes without cables. Secondary aims were to analyze the proportion of new AF cases and related initiation of anticoagulation.

## 2 METHODS

### 2.1 Patients and outcomes

The study was carried out at the Helsinki University Hospital. Ethics approval was obtained from the Ethics Committee of the Hospital District of Helsinki and Uusimaa, and relevant institutional permissions were granted. All patients gave a written informed consent prior to participation as stipulated in the Declaration of Helsinki.

Patients aged ≥50 with recent ESUS (Hart et al., 2017) were prospectively screened and assigned to wear a miniaturized 1‐lead ECG monitoring device for 4 weeks. To meet the criteria of ESUS, patients underwent a diagnostic workup including brain computed tomography (CT) or magnetic resonance (MR) imaging showing findings of a nonlacunar infarction. CT‐ or MR‐angiography was carried out to rule out intracranial and extracranial artery stenosis or other relevant pathology. All patients underwent transthoracic echocardiography (TTE) to exclude major cardioembolic sources of embolism. Selected, mainly younger patients underwent transesophageal echocardiography (TEE). All patients also underwent standard 24‐ or 48‐hr ECG monitoring. Exclusion criteria included diagnosis of AF and other known etiology for the stroke, ongoing anticoagulation therapy at the time of enrollment, and contraindication to anticoagulation therapy.

Patient characteristics, medical history, and medication were recorded. CHA_2_DS_2‐_VASc score was calculated for each patient. Baseline stroke severity was assessed with the National Institutes of Health Stroke Scale (NIHSS). Level of education was graded as low (primary school), middle (upper secondary school/vocational school), or high (university/polytechnic). The primary outcome measure was the proportion of patients successfully completing at least 80% of monitoring with the ECG device. Secondary outcome measures included the incidence of newly diagnosed AF and number of patients to whom anticoagulation therapy was prescribed after new diagnosis of AF.

### 2.2 Monitoring and follow‐up

**Figure 1:**
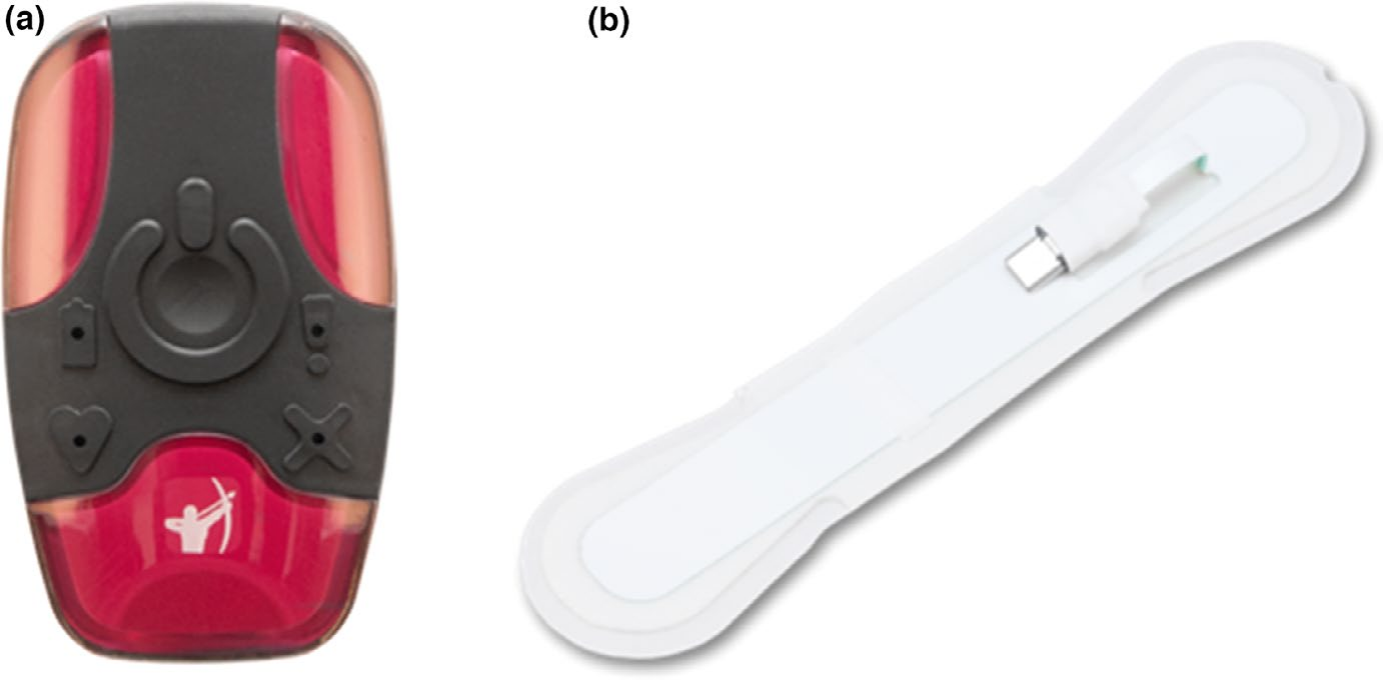
Bittium Faros 180° ECG device (a) and adhesive electrode (b) used in four‐week monitoring

CE‐approved Bittium Faros 180° device (Bittium Inc., Oulu, Finland) with CE‐approved Fastfix (Bittium Inc., Oulu, Finland) adhesive electrodes were used in ECG monitoring (Figure 1). The device has a memory capable of recording ECG for up to 30 days using one channel with 125 Hz sampling rate (Salsone et al., 2016). The optimal placement for the electrode was measured bedside using a mobile ECG application using Bluetooth connection. The application was provided by Bittium Inc. Patients were advised to wear the ECG device continuously for 4 weeks, pausing the recording only when charging the batteries for one hour every three days, taking a shower or bathing (including sauna), or swimming. The adhesive electrode was also changed every three days in order to maintain good data quality. After the 4‐week monitoring, patients returned the device via postal service. Patients received both verbal and written instructions on how to use the device. ECG device has four indicator LEDs (light‐emitting diode) that indicate device functions and possible malfunction. Patients were instructed to monitor indicator lights and contact study personnel in case of malfunction.

The researchers had no financial engagement with the ECG device manufacturer. Devices and software were purchased purely for research purposes with public research funding. The company was not involved in planning of the study, data analysis, or reporting.

All ECG monitoring data recorded by the devices were analyzed retrospectively. Length of monitoring and ECG data quality were recorded. We defined AF as ≥30 s of irregularly irregular R‐R intervals with no detectable P‐waves. A web‐based myDarwin software (Hasiba Medical Inc., Austria) was used to screen ECG recordings, with established Lorenz‐plot based algorithm for the detection of AF (Esperer, Esperer, & Cohen, 2008). The suggested AF episodes were adjudicated by two experienced raters, a cardiologist (T. N.) and a clinical physiologist experienced in Holter interpretation (J. Pi.), blinded to baseline data. We informed the patient's clinical team on the ECG monitoring result immediately after data analysis.

A 3‐month follow‐up was performed, and patient records were reviewed to assess recurrent events, current medication, and functional neurological status (modified Rankin Scale).

### 2.3 Statistical Analysis

Statistical significance was set at *p* < 0.05. All analyzes used SPSS version 24 (IBM Inc., Armonk, NY, USA). Data were reported as *n* (%), mean (± standard deviation [*SD*]), or median (interquartile range [IQR]). Noninterpretable episodes caused by poor ECG data quality were excluded using the quality function of myDarwin software, and only the remaining good‐quality recordings were considered for a quality‐weighted analysis. We compared clinical characteristics of patients with AF with non‐AF patients using the *t* test and chi‐square test. Kaplan–Meier method was used to analyze the rate of AF detection, that is time elapsed since index event.

## 3 RESULTS

### 3.1 Study population and the incidence of AF detection

A total of 83 potentially eligible patients were screened and 62 (75%) gave written informed consent. Five patients were excluded from ECG data analysis: three for lacking compliance for long‐term ECG monitoring due to other comorbidities, one due to incorrectly linked ECG monitoring device (i.e., device could not be matched to correct patient), while one patient lost to follow‐up never returned the device. Of the remaining 57 patients, 52.6% were male with mean age of 64.5 ± 8.2 years.

Baseline characteristics of the study participants stratified by the detection of AF are presented in Table 1. The majority of patients had hypertension, and every fifth had suffered a stroke prior to the index stroke. Patients with diagnosed AF during the study were older and more obese compared to patients without the arrhythmia.

**Table 1:** Baseline characteristics of the study participants (*n* = 57)

Characteristic	All (*n* = 57)	Atrial fibrillation detected		
		No (*n* = 50)	Yes (*n* = 7)	*p* value
Age,years	64.5 ± 8.2	63.6 ± 8.3	70.6 ± 5.1	0.011
Male Sex	30 (52.6)	26 (52.0)	4 (57.1)	0.799
Level of education^a^				
Low	11 (19.3)	10 (20.0)	1 (14.3)	0.129
Middle	24 (42.1)	23 (46.0)	1 (14.3)	
High	21 (36.8)	16 (32.0)	5 (71.4)	
Body mass index	27.0 ± 4.6	26.6 ± 4.6	30.0 ± 3.4	0.039
Score on NIH Stroke Scale	4.3 ± 5.0	4.5 ± 5.3	2.6 ± 2.2	
Hypertension	35 (61.4)	29 (58.0)	6 (85.7)	0.158
Prior stroke	12 (21.1)	10 (20.0)	2 (28.6)	0.602
Active smoking	6 (10.5)	6 (12.0)	0	0.333
CHA_2_DS_2‐_VASc score	3.5 ± 1.2	3.4 ± 1.2	4.3 ± 1.3	0.039
Antiplatelet therapy				
Prestroke	13 (22.8)	12 (24.0)	1 (14.3)	
At discharge	48 (84.2)	43 (86.0)	5 (71.4)	
At 3 months	45 (78.9)	43 (86.0)	2 (28.6)	
Anticoagulation therapy				
Prestroke	0	0	0	
At discharge	9 (15.8)	7 (14.0)	2 (28.6)	
At 3 months	15 (26.3)	8 (16.0)	7 (100.0)	

Note

Data are mean ± standard deviation or *n* (%).

a *n* = 56

**Figure 2:**
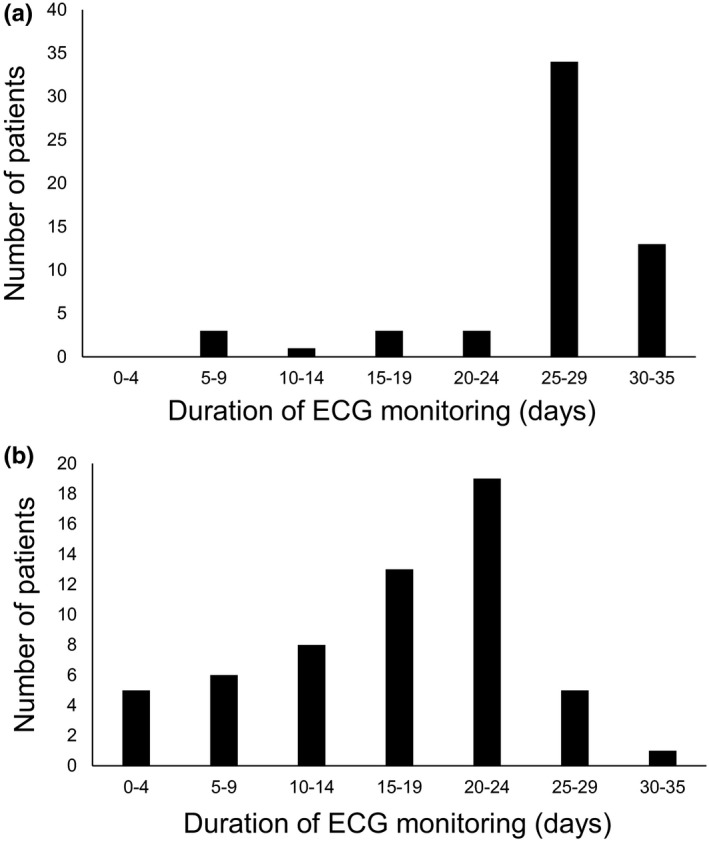
Absolute (a) and quality‐weighted (b) duration of ECG monitoring

Median delay from stroke to the start of monitoring was 8 days (IQR 4–44). Median absolute length of monitoring was 28 days (IQR 26–30), and 19 days (IQR 14–23) in quality‐weighted analysis (Figure 2). A total of 51 (89.5%) patients completed at least 80% of the desired monitoring period in terms of absolute length of monitoring. A total of 18 (31.6%) patients completed at least 80% of monitoring with good quality.

**Figure 3:**
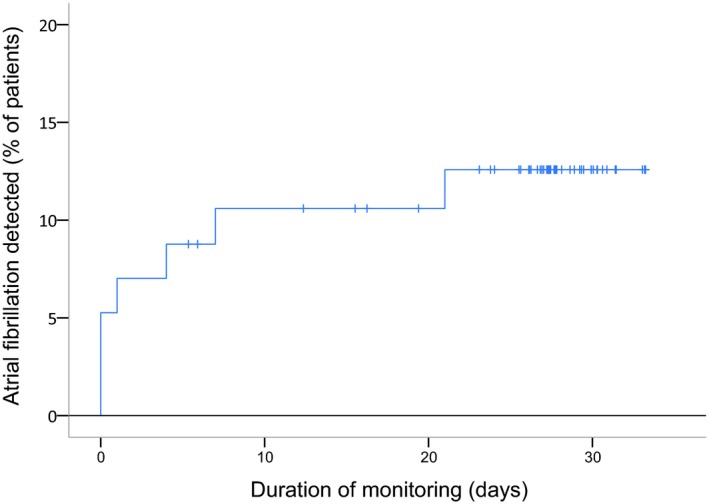
Cumulative detection of atrial fibrillation

New AF ≥ 30 s was detected in 7 (12.3%) patients. A total of 51 AF episodes were detected. We categorized AF episodes based on their symptomatology and duration. Of those diagnosed with AF, three patients reported having symptoms of irregular rhythm, while four patients reported no symptoms. Duration of AF episodes varied from 30 s to 12.5 hr during any of the continuous monitoring periods. Majority (74.5%) of all detected AF episodes were short, lasting from 30 s to 2.5 min. A total of 10 (19.6%) episodes lasting from 2.5 min to 1 hr and 3 (5.9%) lasting from 1 hr to 12 hr were identified. Furthermore, one (2%) episode longer than 12 hr was detected. The two ECG raters reached a full concordance on the AF diagnoses. A great majority of AF episodes detected by the software were false positives as the software often produced dozens to hundreds of false alarms per true AF episode. The median time from monitoring onset to detection of AF was 1 day (IQR 0–7) (Figure 3).

Two (3.5%) patients ceased monitoring prematurely because of skin irritation. No serious adverse effects were reported. Two of the ECG devices were damaged during monitoring due to water damage (shower) and were replaced.

### 3.2 Follow‐up

One patient, in whom AF was not detected during the study period, had a recurrent stroke during the 3‐month follow‐up. The proportion of patients using antiplatelet therapy at the time of discharge from hospital and at the 3‐month follow‐up were 84.2% and 78.9%, respectively (Table 1). Similarly, the proportion of oral anticoagulants at the time of discharge from hospital and by the time of the 3‐month follow‐up were 15.8% and 22.8%, respectively. Of the seven patients with new AF, five had been prescribed anticoagulation therapy before the 3‐month follow‐up and the two remaining AF patients received anticoagulation therapy shortly afterward. Median modified Rankin Scale Score at 3‐month follow‐up was 1 (IQR 0–1) on a scale of 0 to 5.

## 4 DISCUSSION

The main findings of our study are twofold: (a) As many as nine of ten patients with a recent stroke were motivated and wore the device at least 80% of the entire period, evidencing remarkable patient adherence; and (b) four‐week continuous ECG monitoring of ESUS patients using light‐weight ECG recorder and 1‐lead adhesive electrodes resulted in detection of underlying new AF in one out of eight patients.

The detection rate of 12.3% of newly diagnosed AF within four weeks is in accordance with rates previously described in trials studying occult AF detection in cryptogenic stroke (Sposato et al., 2015). Ratio of symptomatic versus asymptomatic AF episodes (3 out of 7 had symptoms) is in accordance with previous studies on prevalence of AF symptoms (Majos & Dabrowski, 2015). Our patients with diagnosed AF were older, more obese and tended to have more hypertension compared to patients without AF. These results are reasonable in terms of knowledge about AF risk factors and epidemiology (Benjamin et al., 2017). The majority (85.7%) of newly detected AF occurred within the first week of monitoring. This finding is in accordance with several other studies, which reported capturing of 50% to 70% of all newly detected AF within the first week of monitoring (Rizos et al., 2010; Sposato et al., 2012).

Our screening population included ESUS patients aged ≥50 years during the study period of two years. The screening period was not entirely continuous, which should be considered as a limitation. Patients completing monitoring were relatively young (mean age 64.5 years), which can imply enrollment bias or suggest that this type of long‐term ECG monitoring is more suitable to younger stroke patients. The included patients also presented with relatively mild symptoms (mean NIHSS score 4.3 points). Our cohort's NIHSS score is, however, comparable to that described in ESUS patients (Hart et al., 2017).

We chose Bittium Faros ECG device because of its capability to store up to 4 weeks of ECG and its particularly compact size and lightweight. Changeable adhesive electrode without cables was chosen in order to make device use as easy as possible for a patient. The protocol and equipment used in the study allowed high patient compliance with nine out of 10 completing at least 80% of the continuous 4‐week monitoring. Median period of good‐quality ECG data was 19 days of the theoretical 28 days. One explanation for this discrepancy could be the fact that the majority of the patients had not used all of the provided electrodes. This will lead to poorer skin contact as the glue used in the adhesive electrodes dries and the plaster wears out mechanically, explaining suboptimal ECG quality. It is also possible that some of the patients altered electrode location when applying a fresh electrode every three days, which may result in worse recording quality than at the primary location, which was optimized. Further analysis would be needed to determine reasons for this finding, although detailed oral and written instructions to users regarding the longevity of an adhesive electrode are clearly needed.

Recent studies of empiric anticoagulation of ESUS patients have not resulted in favorable outcomes. NAVIGATE‐ESUS was preterminated after failing to prove value of rivaroxaban compared to aspirin in secondary prevention of ESUS stroke patients (Kasner et al., 2018). While the results of studies with other anticoagulants are yet to be published, this finding underlines the importance of carefully screening patients for AF.

In conclusion, patients after a recent stroke were highly motivated for screening of AF with a wearable 1‐lead ECG device. Our study showed a relatively large portion of underlying AF that conventional diagnostic methods failed to detect among ESUS subgroup stroke patients, and the AF detection yield increased even after three weeks of recording. The type of monitoring strategy and equipment we used allowed high patient compliance and provided one suitable alternative for prolonged continuous ECG recording in stroke patients. Continuous ECG monitoring allows also effective detection of clinically asymptomatic AF episodes that go unnoticed with, for example, “snapshot” ECG obtained with a handheld ECG devices of smartphone ECG.

## DISCLOSURES

T.L.: None. J.Pu.: Speaker's Honorary: Boehringer‐Ingelheim, BMS‐Pfizer, Bayer, Abbott; Advisory Board: BMS‐Pfizer, Bayer, Boehringer‐Ingelheim, MSD, Portola; Research Collaboration: Nokia Technologies, BcB Medical, Bayer, Vital Signum; Unrestricted Research Grant: St. Jude Medical, Pfizer. A.K. reports personal fees and nonfinancial support from Roche, personal fees and nonfinancial support from Pfizer, personal fees from MSD, outside the submitted work. G.S: None. J.Pi: Research Collaboration: GE Healthcare. S.P: Speaker's Honorary: Bayer, Biotronic, Boston Scientific, Bristol‐Meyers‐Squilbb, Boehringer‐Ingelheim, Medtronic Finland, MSD, Pfizer, St.Jude Medical Finland; Participated in designing of teaching program of pharmaceutical industry companies: Boehringer‐Ingelheim, Bristol‐Meyers‐Squilbb, Pfizer, St.Jude Medical. M.L: None. T.N.: Speaker's Honorary: AstraZeneca, Boehringer‐Ingelheim, BMS, GE Healthcare, Medtronic, Orion, Pfizer, Sanofi, Servier; Advisory Board: Pfizer, Boehringer‐Ingelheim; Research Collaboration: Abbvie, Medtronic.
